# Factors Influencing the Implementation of a Multispecialty Virtual Ward Program in the United Kingdom: Qualitative Exploration of Staff Experiences and Perspectives

**DOI:** 10.2196/75406

**Published:** 2025-06-19

**Authors:** Ian Litchfield, Richard Lewis, Gayathri Delanerolle, Melyda Melyda, Lorraine Harper

**Affiliations:** 1 Department of Applied Health Sciences University of Birmingham Birmingham United Kingdom; 2 Birmingham Health Partners University of Birmingham Birmingham United Kingdom; 3 RQL Consulting Surrey United Kingdom; 4 Queen Elizabeth Hospital Birmingham University Hospitals Birmingham NHS Foundation Trust Birmingham United Kingdom

**Keywords:** virtual wards, qualitative research, digital health, health services research, remote monitoring

## Abstract

**Background:**

The National Health Service (NHS) in England is facing unprecedented demand for hospital services, with virtual wards (VW) being a central tenet of the strategy to manage these ongoing pressures on capacity. VWs combine digital and analog tools, monitoring systems, and teams of multidisciplinary care providers to support patients in their place of residence who might otherwise be cared for in a hospital. Despite virtual ward programs continuing to proliferate in the United Kingdom and across the globe, the models of care that support them are still evolving, and best practices in their design and implementation are yet to be fully established. It is therefore necessary to continue to gather evidence about the influences that shape their design and support their successful and sustained introduction.

**Objective:**

This study aims to explore the experience of staff involved in designing, developing, and delivering VWs as part of the national program, in order to understand the factors that influence their implementation and sustainability.

**Methods:**

Qualitative data were collected through semistructured interviews with staff and senior stakeholders involved in developing, leading, and delivering the virtual ward program within one of the largest integrated care systems in England. Data were analyzed using directed content analysis, informed by the Non-adoption, Abandonment, Scale-up, Spread, and Sustainability (NASSS) framework.

**Results:**

We interviewed 20 participants from clinical and nonclinical roles, including service transformation leads, program leads, physiotherapists, nurses, and consultants. Using the NASSS framework, we identified several key findings: patient context was as important as clinical criteria in determining referral suitability (Condition). Stand-alone digital monitoring solutions with offline capability improved accessibility (Technology). While benefits to patient rehabilitation and hospital capacity were widely understood, concerns over the lack of evidence remained (Value proposition). Clearer messaging about the nature and benefits of VWs was needed for patients and carers, and staff described challenges with remote care and shared responsibility across settings (Adopters). Pre-existing collaborative arrangements helped but varied by specialty (Organizations). NHS targets and metrics of success were considered unrealistic (Wider system). Finally, participants recommended more coherent regional planning that involved consultation with patients (Embedding over time).

**Conclusions:**

If the United Kingdom’s VWs program is expected to move forward, it requires patients, their families, carers, and staff to receive coherent messaging of their responsibilities and benefits. Targeted training and ring-fenced time for staff would help, as would the provision of purposely designed patient-facing technologies. Finally, extended planning and funding cycles are needed to gather robust evidence and refine VWs, ensuring better integration with existing services that incorporate the needs and preferences of patients from various sociocultural backgrounds.

## Introduction

Health care systems in the United Kingdom and across the globe are under growing pressure for a variety of reasons, including aging populations, more complex care demands and treatments, and a corresponding increase in the cost of care [1]. One approach widely expected to help ease that pressure, by avoiding unnecessary admissions, facilitating early discharge, and reducing unplanned visits, is the use of virtual wards (VWs) [2-4]. These combine digital and analog tools and monitoring systems with teams of multidisciplinary care providers to support patients in their place of residence who might otherwise be cared for in a hospital [2,5].

The potential of VWs has driven their implementation across North America [6], Australia [7], Asia [8] and Europe [9]. In the United Kingdom, the National Health Service (NHS) is relying on VWs to help ease the pressure of unprecedented demand for inpatient care [2-4]. The NHS allocated some £500 million (approximately US$ 675 million) with the original intention of creating between 40 and 50 virtual ward beds per 100,000 people by the end of 2025 [10,11]. This implementation was supported via a series of regional VW programs delivered by local integrated care systems and overseen by regional VW leads.

Despite the broad proliferation of VWs globally and the commensurate investment of resources exemplified by the NHS VW program, evidence of their impact and the factors that support the sustainable design and implementation of VWs is still emerging [10,12]. Although patients have consistently expressed a preference for care in their own homes [13] the digital nature of VWs continues to risk excluding certain patient groups, as do broader elements of their social context [14]. From a health service perspective, it is understood that the implementation of VWs can inadvertently and negatively affect other areas of delivery [15,16]. In particular, more evidence is needed around which precise staffing structures, workflows, and models of remote care, are most effective [17]. In supporting those delivering the ward, it is important to understand how they can successfully navigate the challenges of multidisciplinary and interorganizational working, as well as shifts in work practice away from in-person care and toward teleconsultation [17].

To help address some of these continuing gaps in the evidence around VWs, the work presented here explores the experiences of senior decision makers, clinical leads, and those staff delivering multispecialty VW in one of the largest integrated care systems in England. The qualitative data we gathered is presented within an a priori framework purposely developed to explore the implementation of digital health interventions at scale [18]. This has allowed us to offer structured and transferable learning across the VW pathway to inform future iterations of VWs applicable to the United Kingdom and beyond.

## Methods

### Study Design

The work explores the experiences of a range of staff involved in developing and delivering the VW. Data were gathered using semistructured interviews and analyzed using a directed content analysis informed by the Non-adoption, Abandonment, Scale-up, Spread, and Sustainability (NASSS) framework developed by Greenhalgh et al which provides the opportunity for structured learning that is readily transferable across various digital health initiatives [19-21]. The reporting followed the recommended Consolidated Criteria for Reporting Qualitative Studies [22].

### NASSS Framework

**Figure 1:**
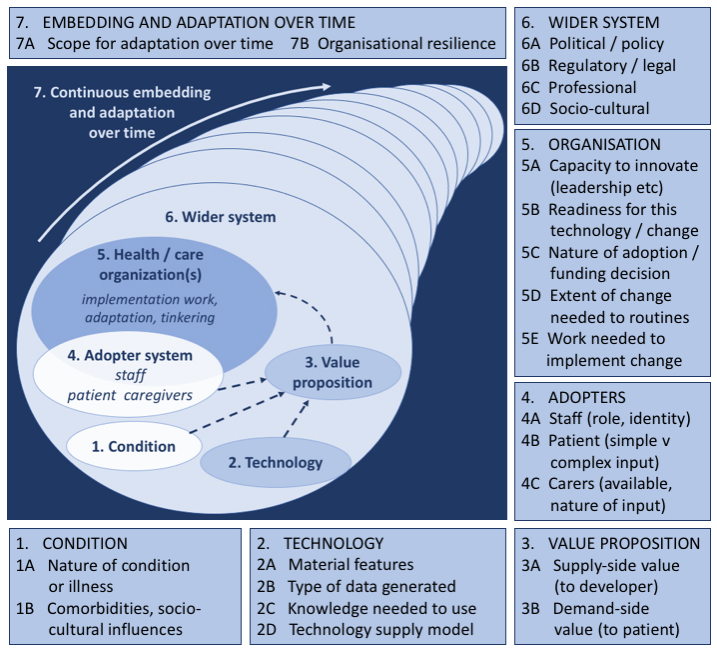
Summary of the Non-adoption, Abandonment, Scale-up, Spread, and Sustainability framework.

The NASSS framework was developed specifically to help plan the implementation and rollout of technology-enabled care programs [20]. Its multidimensional structure allows the systematic identification of the various elements that contribute to the successful delivery of technology-enabled care interventions at scale. The framework consists of seven domains relating to (1) the complexity of the Condition, (2) the nature of the Technology, (3) the Value proposition to stakeholders, (4) the attitudes of and impact on Adopters, (5) the culture and capacity of the Organization, (6) the influence of the Wider system, and (7) the steps needed for the intervention to Embed over time. These domains and the underpinning rationale are summarized in Figure 1 [20] and further described in Table 1 (Abimbola et al [21], Greenhalgh et al [23], Greenhalgh et al [20], and Gani et al [24]).

**Table 1:** Influences on implementation and application to virtual wards presented with the Non-adoption, Abandonment, Scale-up, Spread, and Sustainability (NASSS) framework.

Domain	Definition	NASSS described influences on implementation	Subtheme	Findings from the VW^a^ program
Condition	The conditions or specialties for which the intervention has been designed.	The complexities of the condition	The variation in the clinical requirements of patients and the impact of their social setting	There were notable variations in the model of care between specialties Referral onto the VW was determined by clinical criteria alongside the patient’s digital literacy, connectivity, and level of familial and social support
Technology	The technologies or other innovations that are being introduced. Including hardware and software, a novel protocol or pathway—or some combination of these.	The material properties of the technology	The accessibility of software and hardware to all patients	Patient numbers were restricted by the reliance of the planned patient dashboard on patient-owned technology for example, smartphones, or PCs Accessibility to VWs increased following the introduction of a hospital-distributed, stand-alone tablet with offline capability
Value proposition	The proposed value (financial or otherwise) that the new technology or care model generates.	Better-quality care for patients	The potential improvements in quality and safety of the care delivered	There were widely acknowledged benefits to patients from VWs including greater retention of confidence and independence in comparison to inpatient care
Value proposition	The proposed value (financial or otherwise) that the new technology or care model generates.	Increased service efficiency	The potential improvements in service usage and efficiency	Staff recognized the potential of VWs to increase hospital capacity but expressed reservations around the lack of evidence of their efficacy
Adopters	The intended users of the technology or other innovation. This includes patients and lay people, professionals, administrative and support staff.	Acceptability to patients and family or carers	Ability to meet patient preferences	VWs met patient preference for care at home
Adopters	The intended users of the technology or other innovation. This includes patients and lay people, professionals, administrative and support staff.	Acceptability to patients and family or carers	Patient and carer understanding	Patients and their families or carers appeared to lack awareness of what a VW was, or their benefits
Adopters	The intended users of the technology or other innovation. This includes patients and lay people, professionals, administrative and support staff.	The impact on current roles and professional traditions	Challenges for staff of new working practices	Staff reflected on the issues of sharing responsibilities for a patient across teams and trusts and the need for additional skills in teleconsultations and remote patient management
Organizations	The cultural and organizational characteristics of the organizations involved. This includes structure, capacity, and capability to adopt new ways of working. As well as resources of staff and infrastructure.	Extent of change needed	Readiness for the innovative (VW) pathway	Pre-existing collaborative organizational relationships were supportive but varied by specialty
Organizations	The cultural and organizational characteristics of the organizations involved. This includes structure, capacity, and capability to adopt new ways of working. As well as resources of staff and infrastructure.	Extent of change needed	Ability to incorporate novel (VW) technology	There was a lack of data interoperability between Trusts
Organizations	The cultural and organizational characteristics of the organizations involved. This includes structure, capacity, and capability to adopt new ways of working. As well as resources of staff and infrastructure.	Staff capability and capacity	The work practices that support (VW) delivery	Slow staff recruitment meant the early phases were reliant on temporary workforce structures and flexible staff
Wider system	The regional and national and local context for the introduction of the technology or program.	The impact of national and local policies	Concern over centrally prescribed targets	Staff felt NHS^b^ dictated targets for numbers of VW beds and their occupancy were unrealistic and captured by poorly defined metrics
Wider system	The regional and national and local context for the introduction of the technology or program.	The presence of interorganizational networking	The degree of alignment between health organizations	The importance of ensuring working practices across trusts and between specialties complemented each other The support of the regional NHS team was noted
Embedding over time	The key changes and uncertainties expected to affect the integration of the technology or innovative pathway over the next 3-5 years.	The ability of digitally enhanced care (VWs) to be incorporated into routine care	Standardization of the VW offer	Greater similarity between VWs in different specialties can increase efficiency
Embedding over time	The key changes and uncertainties expected to affect the integration of the technology or innovative pathway over the next 3-5 years.	The ability of digitally enhanced care (VWs) to be incorporated into routine care	The potential of coproduction to shape future services	Patient representatives should be involved in designing or developing the next iteration of VWs

^a^VW: virtual wards.

^b^NHS: Naitonal Health Service.

### Setting/Recruitment

The VW program was delivered by a secondary care trust supported by a local community care trust located in a large, diverse city in the United Kingdom. Both trusts are part of one of the largest integrated care systems in the NHS. Participants were to be purposively sampled to include the NHS regional virtual ward team (nested within the local regional Urgent and Emergency Care team), clinical leads, and those delivering the VW. Those eligible were identified by the VW program Steering Group, made up of representatives from each VW. Staff participants were approached by the VW program leads or senior (virtual) ward staff and those who agreed were then contacted by a researcher via email, offered the opportunity to ask questions about the study and their participation before providing consent and commencing the interview. The intention was to undertake a total of 25 interviews following the recommended estimations of sample size to achieve saturation [25]. The study was approved by the University of Birmingham’s Science, Technology, Engineering, and Mathematics ethical approval committee (ERN_13-1085AP41).

### Data Collection

Semistructured interviews were conducted over the phone or via an online platform (Zoom [Zoom Video Communications] or Microsoft Teams) according to participant preference, between February 2023 and May 2024 inclusive, by IL and RL, who have extensive qualitative experience and backgrounds in applied health research and medical sociology. They were unknown to participants. The interviews followed predesigned topic guides that were informed by previous work that explored the COVID-19 VW program in the United Kingdom [26] and included questions about participants’ roles in developing or supporting the implementation of the VW program, and their personal experiences of delivering the service, including their interaction with regional and national VW teams (the topic guides are summarized in Multimedia Appendix 1). Interviews were digitally recorded and transcribed verbatim by an approved transcription service. The data were then managed using QSR International’s NVivo (version 10; Lumivero) software and Microsoft Excel.

### Data Analysis

The final dataset from the interviews was used in directed content analysis to populate the NASSS framework [20,27]. More precisely, we adopted the “unconstrained matrix” approach suggested by Elo and Kyngäs [27] and Bingham [28], which allows for the development and inclusion of emergent constructs or subconstructs within an established framework. This enabled us to maintain alignment with our established rationale, alongside the systematic categorization of any novel VW practices or constructs that had emerged. Both IL and RL independently coded the same sample of transcripts, fitting the data within the relevant themes of the NASSS framework. Any differences in coding were discussed between the 2 authors, and a consensus was reached. The remaining transcripts were then divided and coded independently. The final allocation of the data within the coding framework was conducted by IL and agreed upon by all authors.

### Ethical Considerations

Ethical approval was granted by the University of Birmingham Research Science Technology, Engineering and Mathematics Ethics Committee (ERN_13-1085AP41). Informed consent was provided by each participant before the commencement of the interview and the subsequent data were pseudonymized and held securely in accordance with the stipulations of the approval.

## Results

### Participant Characteristics

A total of 20 participants were interviewed including one at the national level, 2 with the NHS regional team, 2 with responsibilities for VWs at the Integrated Care System (ICS) level, 7 with the Secondary Care Trust (SCT), and 4 with the Community Care Trust (CCT). Job roles included service transformation leads, program leads, physiotherapists, nurses and consultants. Each interview lasted between 27 and 60 minutes. One participant dropped out after agreeing to be interviewed but identified a replacement of a similar role and seniority.

### Qualitative Data

Staff experiences of delivering the VWs are described below within the 7 domains of the NASSS framework alongside illustrative quotes where staffs are identified by their job category and their role in delivering the service.

### Condition: The Varying Complexities of the Conditions

The design of the VW varied by specialty with significant disparities in referral criteria, staffing models, processes of care, and numbers of patients recruited:

…so respiratory was quite specific in terms of criteria to get onto the virtual ward, so completely focused on COPD early supported discharge … frailty was much more about a wider remit of if somebody is deemed clinically suitable…The increase in occupancy on the virtual wards for frailty have been much better and quicker… whereas respiratory have found it much harder to get going.

P09, Service lead, SCT

Staff went on to describe contextual considerations beyond clinical criteria that influenced referral to the VW, including the patients’ domestic circumstances, their digital literacy and connectivity, and psychological preparedness.

…even though clinically they’ve met the criteria for virtual ward psychologically they are not ready for that.

P18, Service delivery, CCT

There was also the issue of whether patients could independently measure the required physiological parameters:

…you’re thinking “…Really? Would they be able to manage that?” It’s not just being able to tick a box and questions on [patient tablet], it’s being able to actually do a blood pressure, or your SPO_2_ [oxygen saturation levels] or do a temperature.

P19, Service delivery, CCT

### Technology: Material Properties of the Technology

The original offer consisted of using a bespoke digital platform accessed via a PC, tablet, or smartphone. However, only a small number of clinically suitable patients possessed the necessary hardware, internet connectivity, and digital literacy:

…we just aren’t finding the numbers, we don’t have equipment to give them… we haven’t got time to sit and teach them how to use that level of platform, and also we aren’t finding that many patients that are able to use that level of platform.

P05, Service lead, SCT

As a result, the early stages of the implementation relied on telephone and in-person contact to monitor patients. Latterly, a decision was made to purchase a pre-existing purposely designed patient tablet, which improved accessibility as it precluded the need for patients to possess their own hardware, had a simple easily interpretable interface, and offline capability.

The good thing about [Tablet] is that we provide all of the kit, including the tablet, and the tablet is enabled with a SIM card, so there’s no reliance on the patient needing to own any kind of technology, or having broadband or access to the internet.

P14, Service lead, SCT

### Value Proposition

#### Better Quality Care for Patients

Staff understood the benefits to the physical and mental well-being of patients of being cared for in a familiar home environment. This included greater retention of confidence, independence, and physical activity, alongside reductions in hospital-acquired infections.

…inpatient stays are just the worst for anybody… there’s hospital acquired infections, there’s rehabilitation issues … every one day you’re in hospital it’s three days’ worth of rehabilitation…everyone gets pyjama paralysis, they’re losing confidence…they’re not being as active as they were… a lot of people; they struggle when they come out of hospital.

P18, BCHC, Service lead

#### Increased Efficiency

From a service perspective, the potential of VWs to ease pressure on inpatient care by providing a cost-effective alternative was widely recognized by staff. Some, however, expressed concern that policy makers’ desire to increase capacity outweighed considerations of quality:

Virtual ward is a kneejerk reaction…because we need to create capacity. It wasn’t necessarily designed and put together with the first thought of ‘Let’s provide better patient care’, it’s more about ‘We need some capacity!’, and then pitch your care after that.

P15, Service lead, SCT

These concerns were further amplified by the apparent lack of evidence.

…there’s a flaw with the national [VW] programs. They don’t have any evidence…

P02, Service Transformation Lead, SCT

This led one service lead to opine that it felt like a gamble:

There’s never really been a robust evidence base that says that it’s worked. So it felt like a hell of a punt.

P06, Hospital Services Lead, SCT

### Adopters

#### Acceptability to Patients and Caregivers

Staff described patients’ appreciation of leaving the hospital early to be cared for at home, particularly for those who had experienced firsthand the issues of overcrowding:

…the option, to say, ‘Go home from a trolley’ they’re overjoyed! [P16, Service lead, SCT])

More broadly the benefits to a patient’s broader well-being of receiving care at home, such as familiar food and a quieter environment were also recognized:

There’s that ability [of VWs] to be in the comfort of your own home with your comforts around you without the noise of a ward keeping you awake etc, etc, having pets, home food, I think are key for patients.

P09, Programme lead, SCT

There was a lack of coherent messaging around the VW program, including the support provided and its potential benefits. These meant patients were frequently unaware that their hospital discharge constituted a transferal to the “Virtual Ward,” and their family members or caregivers, were suspicious of the hospital’s motivation behind the early discharge:

… [its] the older population that are more nervous of going home, and their carers of having them home, ‘You just want to get mum and dad out, and we’re going to be doing all the personal care, and all you’re going to do is phone them every day?’ The families need to be reassured as well.

P18, Service lead, CCT

#### Impact on Current Roles and Professional Traditions

The delivery of VWs required clinicians to adopt new ways of working. This involved the handover of recovering patients to other care teams in the community counter to usual care where clinicians retained direct responsibility until discharge (“…clinicians have found that really, really, difficult… [for] the old school nurses and clinicians among us it doesn’t sit well…” [P18, Service lead, CCT]). These changes to work practices also included an increased reliance on remote monitoring and teleconsultation requiring a slightly different set of skills:

Clinicians are concerned about that [remote consultation], because they’re so used to doing a face-to-face, even to be able to do a few ‘obs’ [observations] on a patient, just to get a bit of information and talk to them - it’s different doing that by telephone, or virtually... It’s a different mindset…and different decision-making skills…

P19, Service delivery, CCT

### Organization

#### Extent of Change Needed

Participants described how the respiratory VW benefited from strong, pre-existing relationships between local partners. However, for those delivering the frailty VW, such collaborative pathways were less established, leading to uncertainty in where clinical responsibility lay:

… you have a consultant telling you - or advising you - to ‘halve the medication’… ‘double the medication’. Now that’s great as the consultant, and I don’t question the validity in her decision-making, but…we’re always taught as nurses you don’t just do as you’re told, … So, I was very clear to the staff to [write] that…’This is the conversation, The advice given was this, Therefore I shared this information with the patient, and they agreed’ … not for blame, but to share that responsibility equally...

P17, Service delivery, CCT

Familiar challenges of data interoperability emerged [29], with staff describing how patient data were added manually to spreadsheets in community settings:

They [IT systems] need to be better, because we’re still tending to rely on maybe a spreadsheet or something to collate things on.

P19, Service delivery, CCT

#### Staff Capacity and Capability

It was noted that delays in recruiting to the VW, in part due to uncertainty over short-term funding cycles led to interim staffing solutions to deliver the service, with staff being sometimes reluctantly co-opted to work on the VW:

…there’s actually nobody employed at the moment - or hardly anybody - to do virtual wards, because it being a new project. So, they’re just beg and borrowing people from other parts to do it.

P19, Service delivery CCT

### The Wider System

#### Impact of National and Localized Policies

Participants described what they considered to be NHS’s unrealistic expectations of how quickly it expected VWs to be up and running (“…it was forced through, pushed through at speed” [P03, Clinical Lead, SCT]). The way NHS defined the metric of bed usage (ie, proportion of VWs occupied) was considered problematic as it failed to account for how many unique patients were using the beds and what the overall length of stay was per patient. They also felt the number of beds that the NHS was expecting sites to deliver was unrealistic, amidst suspicions that some sites were gaming the system to meet these targets:

The numbers were bonkers…the numbers that were stated [by NHS] … they were suggesting within 18 months you’d be opening up the size of a district general hospital!

P06, Hospital Services, SCT

#### Presence of Interorganizational Networking

There was an understanding that the teams associated with the different specialties across the CCT and SCT had independently developed or adapted different processes when the VW program began. However, to be sustainable, the VW teams would face a choice to align these where appropriate or otherwise leave them in a place where reflective of localized context (“... it’s knowing where it’s worth trying to align and where it’s worth actually accepting that it might be done a little bit differently” [P06, UHB, Service lead]). At a system level, program leads described the constructive relationship with the NHS’s Regional VW team, who acted as advocates for the local VW offer:

…on a number of occasions [the regional link person] would actually on our behalf go back [to the national team] and say ‘I don’t think this is the right approach’, or ‘actually, the way they’re approaching it and the numbers they’re putting against this, is right’…

P02, Service Transformation Lead, SCT

### Embedding Over Time

#### Ability of VWs to Be Incorporated Into Routine Care

Pre-existing pathways for respiratory and frailty care were very different, leading to the community team accommodating 2 different approaches to VWs. As a result, steps are underway to develop a more generic community component:

If I could give one bit of advice over a year ago to everybody starting virtual wards is don’t start it in one specialty… I found it quite hard to standardise the operational side. Because… and I suppose it was timing, we had to setup different ways of delivering stuff in different specialties at different times…

P18, Service lead, CCT

#### Potential of Coproduction to Shape Future Services

Staff described how patients had not been involved in the development of the VW program (“…from processes that I have been involved in… you’d have a service user present…To my knowledge that hasn’t been done” [P13, Service lead, SCT]). Nor were there joined-up consultations across the ICS as to how VWs and in-patient wards could be developed in partnership to meet growing demand:

I know that on the acute side they will have done a calculation that says we need another 100 acute beds in [Location]…You just know intuitively that virtual wards is part of that solution, but the plans for how many acute beds we need and how many virtual ward beds need they are very separate, we’re not doing this as a joint enterprise…

P14, Service lead, SCT

## Discussion

### Principal Findings

The rapid implementation of a VW program across a large ICS in England provided an opportunity to gain valuable insight into the practicalities of delivering VWs at scale and speed. In considering our findings within the domains of the NASSS framework (as summarized in Table 1), we found that: patient context was as important as clinical criteria in determining suitability for referral (Condition); stand-alone digital monitoring solutions, with offline capability, increased accessibility (Technology); the benefits to patient rehabilitation, and hospital capacity were widely understood but concerns remained over the lack of evidence (Value proposition): for patients and carers there was a need for more prominent messaging of the nature and benefits of VW; for staff the need to overcome the challenges presented by remote care and sharing responsibility across trusts (Adopters); pre-existing collaborative arrangements helped, but their maturity varied by specialty (Organizations): NHS targets, and their metrics of success were considered unrealistic (Wider system); finally, there were recommendations for more coherent regional planning that involved consultation with patients (Embedding over time). Below we set these findings within the context of existing literature on VWs and similar models of technology-enabled care.

### Limitations

The NASSS framework proved a capable means of analyzing and presenting staff experience of a significant VW offer to a large diverse patient population. However, at the time of data collection, only the “early supported discharge” element of the VW was used, though admission avoidance (ie, the referral from primary care directly onto the VW) is now an equally important element of this and other VW models. Although only one ICS was used in the study, the patient population it serves is diverse, consisting of a range of cultures and socioeconomic status reflective of the broader population of the United Kingdom. The wide range of roles and responsibilities of participants provided a thorough health service perspective though the numbers recruited were lower than expected, particularly of those delivering the service. This is likely attributable to the pressure staff were underdelivering the VW in the early phase without sufficient numbers dedicated to the role. To support participation rates, data collection was undertaken over a period of 16 months, which also allowed us to capture the perspectives of staff toward the end of this period as they witnessed the service mature and the expectations of the NHS evolve. Data saturation was reached in smaller numbers than anticipated, which could be explained by “consensus theory,” where “experts” with shared knowledge about a tightly defined topic are more likely to exhibit common values [30]. Despite this, future research should endeavor to recruit more staff with direct experience in delivering VW services. Using the unconstrained matrix approach meant that all of the data was capable of being mapped onto the NASSS framework. However, we expect that VWs being delivered in other contexts will uncover slightly different findings within each of the domains of the NASSS. It is recognized that the planned exploration of patients, caregivers, and the broader health care system will provide a more complete understanding of the overall impact.

### Comparison With Previous Work

#### Condition

VWs are typically referred to as a singular care offer by policy makers, commissioners, and service leads [17]. However, our participants described notable variations in the way VWs were used and delivered between specialties: these included variations in the use of specialty clinical staff, digital tools, level of monitoring, as well as the length of time they were expected to remain on the ward. This meant that the patients deemed appropriate for referral to the VW varied in the nature and severity of their illness or condition, as did the requirements of the staff and surrounding services. These clinical variations in the demands and expectations of VWs are becoming more widely recognized and are beginning to shape the definition and commissioning of VWs globally. What is not explicitly described in the literature around VWs is the need for referrers to understand the social context of patients [2,5,8,12]. This context goes beyond the challenges of internet connectivity and digital literacy that are typically linked with a patient’s suitability for VWs [31] to include the overlooked tasks of meal preparation and hygiene. These factors were equally important when considering referrals, although these have been less frequently considered by those commissioning VWs in the United Kingdom [16,31,32].

#### Technology

The digital transformation of health care, including the growing use of VWs envisioned by policy makers in England and further afield, is predicated on the expectation that care pathways will be reliant on their digital component [33-35]. However, participants described how the VW existed as a hybrid service which was at least as dependent on telephone and in-person contact as digital technologies, as also observed in multiple examples of VW services across the globe [2,8,17,36-39]. There is a growing understanding of the need to retain human interaction in a range of digital care services, in order to support patient engagement and foster trust [40,41]. However, in the context of VWs and work is needed to more precisely understand exactly what the human component looks like, and how the need for lesser or greater human interaction might be assessed across a range of patients.

The potential of digital health technologies to be used by digitally literate and connected patients is widely understood [42,43]. Despite this, the sophistication of the original monitoring platform and its reliance on patient-owned technology, limited the number of patients that could use it. Latterly, the adoption of a purposely designed patient tablet that followed recommendations for the design of digital health tools, such as a single login, off-line capability, and an easy-to-navigate dashboard, meant the VW became a viable option for a wider range of patients [44-46]. The gap between design and the capability of the end user in digital health technologies can be overcome by using the lived experiences of a diverse range of patients in digital co-design to ensure the solution is directly compatible with diverse lifestyles and cultures, and existing devices, connectivity, and health and digital literacies [47].

#### Value Proposition

Participants recognized the need for the ICS to create additional in-patient capacity in their busy secondary care facilities [1], they also understood the previously reported benefits to patients of VWs, such as reducing secondary infection rates and rehabilitation times [13,48,49]. Despite this broad recognition of the potential of VWs, staff described concerns that have been recorded previously by staff delivering other examples of VWs, that there was a rush to implementation in advance of sufficient evidence of their efficacy and safety [17]. In particular, concerns over the safety of VWs are longstanding, where they are to be used with complex patients or otherwise high levels of clinical uncertainty [5,50-57]. This work has again highlighted the apparent disconnect between the confidence of those commissioning VWs more circumspect attitudes of those delivering them. Knowing that clearly communicating evidence is the key to successful change management then those advocating for or commissioning VWs service might begin by providing more coherent messaging around existing evidence on the efficacy and safety of VWs if these long-standing concerns are to be overcome [58].

#### Adopters

Participants described the change in work practices necessitated by VWs which included sharing responsibility across community teams and hospital-based staff. It was also noted that teleconsultations require a different set of skills than those needed for in-person consultations. This has been widely acknowledged in previous iterations of VWs [5,59-61] and has led to the creation in some countries of specialized training for those employed in VW programs [62]. Yet implementing such training, particularly for pilot programs like this, where staffs are seconded to the service remains challenging [63].

Despite not specifically referring to the VW, the positive patient response to being discharged early to be cared for at home has been witnessed at other VW services, both in this particular NHS VW program [64-69], as well as a range of VW offers in other countries [13,70-72]. However, some of their families and carers were suspicious of the rationale behind “Virtual Wards” amidst concerns they were assuming additional responsibility on behalf of the hospital. The lack of awareness and misplaced assumptions might be attributed, at least in part, to the lack of consistent patient-facing messaging known to play a key role in successfully implementing other technology-enhanced health services [73]. The lack of clear communication with patients has previously affected their level of engagement with the digital component of similar supported self-management initiatives, adversely impacting clinical outcomes [60,74-76].

#### Organization

Interprofessional collaboration is key to sustaining VWs [77]; yet, participants described uncertainties in where clinical responsibility lay between community staff and hospital-based consultants, an issue witnessed previously in VW services [39,59]. In the United Kingdom, the NHS’s guidance for the clinical leadership of VWs specifically recommends that such responsibilities are openly negotiated and clearly defined between teams working in different settings [78,79]. These conversations did take place at a senior level, but the challenge of questioning decisions made by senior staff remained, and though these issues are not restricted to VWs they appear to be exacerbated by the physical dislocation of community staff [80].

The lack of data interoperability that surfaced in this study led to patient data being entered manually despite the associated risk of error [81]. These data interoperability issues are widespread in the NHS [82-85]. In an attempt to minimize the impact on VWs the NHS has produced specific recommendations for the procurement of digital platforms for VWs [86]. However, with uncertainty over the long-term future of VWs it is unlikely sites will purchase potentially expensive systems to manage VWs meaning stop-gap solutions to data entry will likely continue [87].

In the United Kingdom and globally, it is understood that the capability of adopting innovative, technology-enhanced care is dependent on the number and experience of the existing workforce [88-90]. Reflecting this, our participants described the impact of delays in recruitment to the VW program, which meant some were moved at short notice, without training, and often alongside existing roles. This experience echoes broader concerns that shortages in appropriately trained or experienced NHS staff could derail the intended proliferation of VWs in the United Kingdom [91,92]. Some of the recruitment delays were due to the hesitation of employing staff for a VW program that had secured only short-term funding. It’s recognized that short-term funding cycles are inhibiting long-term service improvement in the NHS [93], including VWs [16]. In the United Kingdom, longer-term planning is inhibited by the differing political priorities of successive governments meaning that 10- or even 5-year plans are altered or discarded before completion, the result is for growing recommendations of cross-party agreement on planning and funding cycles spanning 30 years or longer [94].

#### Wider System

In the United Kingdom, centrally mandated and highly reductive targets have been previously criticized for distorting the implementation of complex system-wide initiatives in the NHS [95]. Specifically regards this pilot, participants questioned the feasibility of creating the expected number of occupied VW beds and the metrics used to assess that capacity [12,95]. More achievable numbers provided by senior ICS VW staff were fed back to national leads via the regional NHS VW team with the result that targets were readjusted, suggesting that in the future more honest conversations with central commissioners might produce consensual targets that better accommodate local context [16,96,97].

#### Embedding Over Time

Participants described how the considerable variation in the design of each VW presented difficulties for community staff delivering the service, in line with previous recommendations for the sharing of common standards and processes in VW offers [13]. At this study site service leads intend that the next phase will involve a more generic model of VWs. There was also a lack of patient input in the early stages of designing the VW program. It is widely understood that patient involvement can help bridge the gap between technology and patient-centered care [98-101], and the next iteration may benefit from earlier and more structured patient involvement, not least in the support of decisions on the procurement of patient-facing technologies, as well as joined up conversations on capacity across the ICS.

### Implications for Practice

This study highlights several longstanding challenges to VWs that remain unresolved and merit careful thought for future iterations. These are summarized in Table 2 alongside a series of questions that might be considered by commissioners, senior decision makers and others involved in the design and delivery of the next generation of VWs. They include the need for a more structured and consistent approach to assessing the social context of patients and the additional support needed for their successful referral to the VW; how digital literacy and connectivity might be more consistently captured before referral; to what degree has existing evidence been used to refine the VW offer and how has this been communicated to staff to support their engagement; the extent to which there has been appropriate communication and messaging on the nature and benefits of VWs and the managing of expectations of patients and their families or carers; the system and processes put in place to support training of VW staff; and finally, to what extent has the planning cycle included the goals and objectives of the surrounding system and incorporated the experience of staff and patient end users?

**Table 2:** Considerations for future virtual wards implementation in the National Health Service.

NASSS^a^ domain	Theme from the evaluation	Questions to ask before proceeding
Condition	An understanding of social context of individual informs the decision to refer to VW^b^	How is social context formally captured at the time of referral? How are decisions made on adequacy of the available support from informal care providers (eg, family caregivers) and whether additional support is needed?
Technology	Issues with suitability of smartphone-based technology	Is there an understanding of the digital literacy, internet connectivity of individuals? Have patients been consulted on the appropriateness of the chosen technology?
Value proposition	Concerns amongst staff that expansion of VWs driven by concerns over capacity (with a lack of evidence of safe, efficient and cost-effective care)	Has the latest (NHS^c^) evidence been used in designing or refining the VW service? What does the staff messaging look like around the rationale for introducing VWs?
Adopters	Staff: Concerned over change in roles and shared responsibility across settings	Have staff been engaged in the development and delivery of VWs? Are responsibilities of various roles defined and understood by all, particularly between Trusts?
Adopters	Patients: Lack of understanding of being on the VW including its safety and rationale; issues over inclusivity	What does the messaging for patients and families or carers look like? Are the benefits and safety procedures routinely explained (and understood)? To what extent are patients from underserved populations being catered for and how is this being determined?
Organizations	There were differences in the experience and training of the staff delivering VWs	Has specific training for VWs been considered? How capable are the staff of managing IT systems and conducting teleconsultations?
Wider system	A lack of coherence in long-term planning and the impact of longstanding challenges to resource	How far reaching is the planning cycle and to what extent are all Trusts and settings involved?
Embedding over time	Lack of standardization of the service and a lack of coproduction impacted the ability to incorporate the service into usual care.	Are there plans for co-production of the next iteration of the VW? Are a range of patients involved?

^a^NASSS: Non-adoption, Abandonment, Scale-up, Spread, and Sustainability.

^b^VW: virtual wards.

^c^NHS: National Health Service.

### Conclusions

VWs appear to be a lasting component of health care delivery, as evidence of their optimization continues to emerge. This study has provided valuable insight into the experience of a range of staff delivering the latest iteration of VWs in the United Kingdom. Much of what emerged has been witnessed in previous VW initiatives globally, though participants also described less widely reported phenomena that, taken together, have produced some broadly transferable considerations for VWs to become a more sustainable and equitable model of care.

## Appendix

Multimedia Appendix 1 Additional material.

## Abbreviations

CCT: Community Care Trust
ICS: Integrated Care System
NASSS: Non-adoption, Abandonment, Scale-up, Spread, and Sustainability framework
NHS: National Health Service
SCT: Secondary Care Trust
VW: virtual wards

## Data Availability

The datasets generated or analyzed during this study are not publicly available for reasons of confidentiality but are available from the corresponding author on reasonable request.
