# Supplementary material for: Factors Influencing the Implementation of a Multispecialty Virtual Ward Program in the United Kingdom: Qualitative Exploration of Staff Experiences and Perspectives
Source: J Med Internet Res. 2025 Jun 19;27:e75406. doi: 10.2196/75406 (PMC12226784; doi:10.2196/75406)
Supplement: Multimedia Appendix 1 [file jmir_v27i1e75406_app1.pdf]

Multimedia Appendix: Summary Topic Guides

This is a Multimedia Appendix to a full manuscript published in the J Med Internet Res. For full copyright and citation information see http://dx.doi: 10.2196/75406

Summary topic guide- Service leads

1. Can we start with a description of your current role?
   (Prompts included)
   Length of time in post,
   Key responsibilities)

2. How did the model of VW for [condition] originate and how did it develop over time?
   Who led the development of the model?
   Has any learning from VWs elsewhere been incorporated?
   What was the nature/extent of partnership working to design/deliver the model?

3. What are the aims of the VW and its main features?
   What is your understanding of the patient groups being served

4. What are the main goals/outcomes of the service/model?

5. What are the key processes involved in the VW model?

6. Can you share your thoughts about patient safety concerns and/or near misses that have occurred since the service began?

7. Have there been any occasions of patients refusing treatment and/or dropping out?

8. What impact, if any, has the introduction of the service/model had on the following:
   Patients and their management of [condition]
   Tackling health inequalities and/or reaching high risk populations
   Delivery of the service within your own organisation
   The wider health and care system

9. What are the factors that act as barriers and facilitators in the design and implementation of VW?

10. What is the current staffing arrangement used to deliver your model?
    Number of staff/ pay band/grades
    Any new additional staff been recruited
    Redeployment of staff working elsewhere within the organisation

11. How is patient data linked across systems?
    Who is able to access these data?

What is your opinion on the quality of the data?
What data or information, if any, would you have liked to have collected

12. What are the lessons learnt from implementing the VW?

Interview topic guide_ Staff delivery

1. Can we start with a description of your current role?
   Length of time in post,
   Key responsibilities

2. What are the aims of the VW and its main features?
   What is your understanding of the patient groups being served

3. May you describe your experience of delivering VW specific to your role?
   Referral processes (variation by age, ethnicity, deprivation)
   Patient triage
   Patient information and training
   Patient monitoring (what was monitored and how)
   Mechanisms used for patient data reporting (i.e. app, paper-based)

4. Can you share your thoughts about patient safety concerns and/or near misses that have occurred since the service began?

5. Describe how you felt when you learned about the use of the technology to support patients in the home?
   Confidence about their own technological/digital literacy
   Previous experience of using a new technology to treat patients
   Have their attitudes changed towards the use of digital platforms since working on the VW?

6. Can you describe the training you have received to explain and deliver the VW to patients? What further training would you like?

7. What skills, from your previous/existing role, have been useful when delivering VWs to patients?

8. Describe the experience of working with new staff or across Trusts
   Challenges and tensions (e.g., communication with new colleagues)

9. Can you describe the nature of support and guidance you received (if any) during the set up and delivery of the service from within your organisation?

10. Can you describe the nature of your engagement with patients referred to the VW?

Did you have trouble accessing any patient groups? Has there been any tailoring of the service to meet specific needs/requirements?
Do you feel patients and carers received all of the necessary information? Do you feel they understood the information?
Do you feel that you gathered enough information from your patient and carers in relation to their wider social circumstances to understand how best to use remote monitoring for them?
Did any patients appear anxious/need reassuring at any stage?
How would you describe your experience engaging with family members and/or carers of patients?

11. What are the lessons learnt from implementing the VW?
